# Altered oscillatory cerebral blood flow velocity and autoregulation in postural tachycardia syndrome

**DOI:** 10.3389/fphys.2014.00234

**Published:** 2014-06-23

**Authors:** Marvin S. Medow, Andrew T. Del Pozzi, Zachary R. Messer, Courtney Terilli, Julian M. Stewart

**Affiliations:** ^1^Departments of Pediatrics, The Center for Hypotension, New York Medical CollegeValhalla, NY, USA; ^2^Departments of Physiology, New York Medical CollegeValhalla, NY, USA

**Keywords:** cerebral blood flow, postural tachycardia syndrome, transfer function analysis, mean arterial pressure autospectra, mean cerebral blood flow velocity autospectra, vasomotion

## Abstract

Decreased upright cerebral blood flow (CBF) with hyperpnea and hypocapnia is seen in a minority of patients with postural tachycardia syndrome (POTS). More often, CBF is not decreased despite upright neurocognitive dysfunction. This may result from time-dependent changes in CBF. We hypothesized that increased oscillations in CBF occurs in POTS (*N* = 12) compared to healthy controls (*N* = 9), and tested by measuring CBF velocity (CBFv) by transcranial Doppler ultrasound of the middle cerebral artery, mean arterial pressure (MAP) and related parameters, supine and during 70° upright tilt. Autospectra for mean CBFv and MAP, and transfer function analysis were obtained over the frequency range of 0.0078–0.4 Hz. Upright HR was increased in POTS (125 ± 8 vs. 86 ± 2 bpm), as was diastolic BP (74 ± 3 vs. 65 ± 3 mmHg) compared to control, while peripheral resistance, cardiac output, and mean CBFv increased similarly with tilt. Upright BP variability (BPV), low frequency (LF) power (0.04–0.13 Hz), and peak frequency of BPV were increased in POTS (24.3 ± 4.1, and 18.4 ± 4.1 mmHg^2^/Hz at 0.091 Hz vs. 11.8 ± 3.3, and 8.8 ± 2 mmHg^2^/Hz c at 0.071 Hz), as was upright overall CBFv variability, low frequency power and peak frequency of CBFv variability (29.3 ± 4.7, and 22.1 ± 2.7 [cm/s]^2^/Hz at.092 Hz vs. 14.7 ± 2.6, and 6.7 ± 1.2 [cm/s]^2^/Hz at 0.077Hz). Autospectra were sharply peaked in POTS. LF phase was decreased in POTS (-14 ± 4 vs. -25 ± 10 degrees) while upright. LF gain was increased (1.51 ± 0.09 vs. 0.86 ± 0.12 [cm/s]/ mmHg) while coherence was increased (0.96 ± 0.01 vs. 0.80 ± 0.04). Increased oscillatory BP in upright POTS patients is closely coupled to oscillatory CBFv over a narrow bandwidth corresponding to the Mayer wave frequency. Therefore combined increased oscillatory BP and increased LF gain markedly increases CBFv oscillations in a narrow bandwidth. This close coupling of CBF to MAP indicates impaired cerebral autoregulation that may underlie upright neurocognitive dysfunction in POTS.

## Introduction

Orthostatic Intolerance (OI) is defined by signs and symptoms such as lightheadedness, tachycardia, hypotension, hyperpnea, headache, fatigue, cognitive deficits, exercise intolerance and nausea (Low, 1993; Suarez et al., 1999) while upright relieved by recumbence (Robertson, 1999). Some OI findings, such as nausea and sweating result directly from changes in peripheral autonomic activity (Okamoto et al., 2012), while findings such as lightheadedness, and cognitive loss pertain to central nervous system (CNS) function (Ross et al., 2013; Shanks et al., 2013). POTS is defined by chronic OI associated with excess upright tachycardia (Streeten et al., 1988; Streeten, 1990; Schondorf and Low, 1993; Low et al., 1995; Jacob et al., 1997). POTS patients often describe CNS symptoms of impaired awareness, mental confusion, lightheadedness, mental fatigue, and cognitive deficits especially in working memory (Ross et al., 2013) which are almost always present when upright and compel patient recumbence to improve symptoms.

We hypothesized that orthostatic reductions of cerebral blood flow (CBF) in POTS due to increased cerebral vasomotor tone (Dewey et al., 1974) impair neuronal activation (Sabri et al., 2003) due to diminished cerebral autoregulation. Although on average CBF decreased more in POTS than controls (Ocon et al., 2009) more recent studies showed that large abnormal decreases in mean CBF occurred during orthostasis in a subset of POTS patients in whom a significantly decreased central blood volume during initial orthostasis triggered hyperpnea, hypocapnia, and sympathetic activation (Del Pozzi et al., 2014). In these hyperpneic POTS patients, reduced CBF likely compromised CNS function resulting in increased cerebral oxygen demand, neuronal excitability, and continued sympathetic activation (Laffey and Kavanagh, 2002; Del Pozzi et al., 2014). In contrast, smaller transient decreases in AP and central blood volume known as “initial orthostatic hypotension” (Stewart, 2013) occurred in healthy volunteer control subjects soon after standing but had no enduring effect on CBFv (Thomas et al., 2009). Nevertheless, “postural hyperpnea” falls short as an overall explanation for orthostasis-induced diminished CNS function because it does not occur in a majority of POTS patients.

The effects of low CBF on CNS function were examined in experiments using graded incremental head-up tilt, and executive working memory evaluation using N-Back testing as an objective measure of cognitive impairment. In these determinations, sudden decreases in CBF did not occur in either POTS or control subjects, and mean CBF, while decreasing with tilt angle, was not different for POTS and controls (Stewart et al., 2012). Executive memory function however was progressively impaired in POTS with increasing angle of tilt.

**Figure 1:**
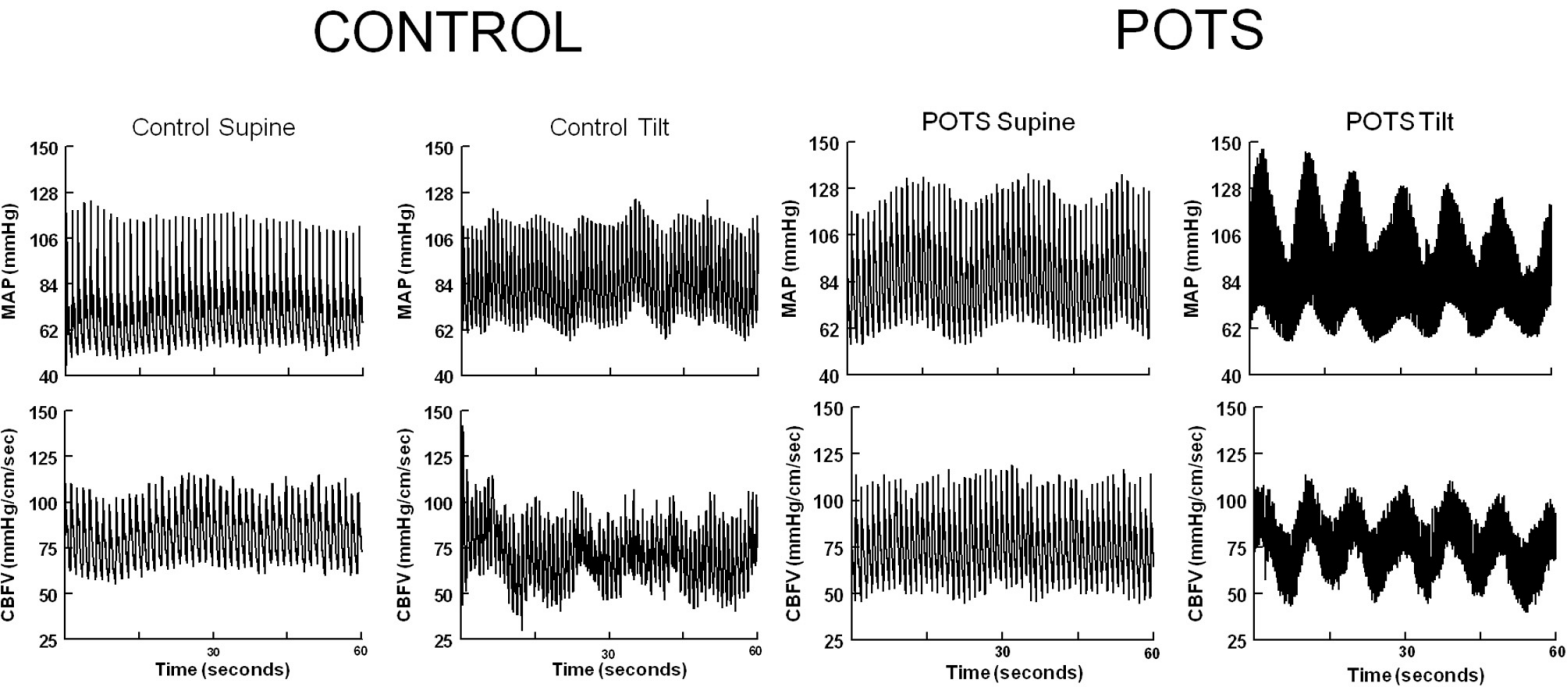
**Representative supine and upright control and POTS phasic arterial pressure (AP) and cerebral blood flow velocity (CBFv) in the time domain**. With tilt, oscillations in arterial pressure intensify in POTS, less so in control and at a lower frequency. Oscillations in arterial pressure are synchronous with oscillations in CBFv in POTS.

In the absence of consistent differences in CBF characteristics that could distinguish POTS from control subjects during orthostasis, we examined the dynamic properties of CBF. We observed that increased oscillations in arterial pressure (OAP) were very evident in POTS patients when upright and appeared to synchronize with increased oscillations in CBF (OCBF) as shown in Figure 1. Similar increases in CBF oscillations have been described previously, but in a more heterogeneous cohort of POTS patients (Schondorf et al., 2005). While these oscillations are seen in all of our POTS patients, they were more distinguishable in patients without postural hyperpnea. For this investigation therefore, we chose to enroll POTS patients in whom hyperpnea was absent.

We tested the hypothesis that there is increased oscillatory CBF in POTS patients without hyperpnea, compared to healthy volunteers. If coupled to OAP, OCBF could be increased either because of increased OAP or because of increased coupling. We therefore also investigated whether OAP was also increased and whether its coupling to OCBF was enhanced in POTS patients while upright.

## Materials and methods

### Subjects

We enrolled 12 of 17 consecutive POTS subjects excluding 5 subjects who developed postural hyperpnea during testing. POTS subjects were 16–29 years old (median age 20.8 years, 9 female, 3 males) with POTS defined by standard criteria: In brief, POTS was defined by chronic day-to-day symptoms of OI plus an excessive increase in sinus heart rate when upright without hypotension (Schondorf and Low, 1993). All patients had symptoms for 6 months or more. Excessive tachycardia was defined in adults (> 19 years) by a sustained increase exceeding 30 bpm or a HR >120 bpm during a 10 min tilt (Low et al., 1995). Concurrent OI symptoms during testing were necessary. Because higher HR changes on upright tilt are observed in healthy children, we used a larger HR increment during tilt of at least 40 bpm (Singer et al., 2012) to diagnose POTS in young people ≤19 years old.

POTS was identified during upright tilt table testing to 70° by signs and symptoms of OI and an excessive increase in HR within 10 min of head-up tilt (HUT) (Low et al., 1995; Raj, 2006; Medow and Stewart, 2007). No other medical problems could explain these signs or symptoms. Normocapnia in POTS subjects was defined as an end-tidal CO_2_ (ETCO_2_) between 35 and 45 mmHg both supine and during 70° HUT and is the range of ETCO_2_ observed in our healthy volunteers.

Nine consecutive healthy volunteers were enrolled as control subjects aged 17–27 years old (median age 21.4, 6 female, 3 male). Healthy control subjects were defined as individuals having no previously known medical conditions, free of systemic illness, with a normal physical exam and electrocardiogram, having never experienced OI of any type, including orthostatic hypotension, POTS or syncope.

Trained athletes, bed-ridden individuals, and individuals who used nicotine-containing products were excluded from enrollment. All subjects were required to refrain from all medications for at least 2 weeks prior to the study with the exception of contraceptive medications. All subjects were required to stop ingestion of xanthine-, caffeine-, or alcohol-containing substances 72 h prior to study. A light breakfast was permitted on testing day if eaten 2 or more hours prior to testing.

The Institutional Review Board of New York Medical College reviewed and approved this protocol. Each subject received a detailed description of all protocols and was given an opportunity to have their questions answered. Signed informed consent was obtained from all participants or their guardian.

### Instrumentation

All subjects were instrumented in a similar fashion by the same operators. Height and weight were measured. During instrumentation, all subjects lay supine on an electronic motorized tilt table (Colin Medical Instruments Corp., San Antonio, TX) with a footboard. Beat-to-beat blood pressure was monitored using finger arterial plethysmography (Finometer; FMS, Amsterdam, The Netherlands) on the right middle or index finger and corrected for tilt angle. Finometer data were calibrated to brachial artery pressure. Modelflow® software was used to estimate beat-to-beat relative changes in cardiac output (CO) calibrated against Innocor CO (Innovision, Denmark). A single lead ECG measured HR. A nasal cannula connected to a capnograph with a pulse oximeter (Smiths Medical, Waukesha, WI) measured ETCO_2_ and O_2_ saturation. Transcranial Doppler (TCD) (Neurovision; Multigon, Yonkers, NY) measured CBFv of the left middle cerebral artery (MCA) using a 2 MHz probe fixed to the subject's head by a custom-made headband. All analog signals were digitized at 200 Hz with custom signal processing software and analyzed off-line.

### Protocol

All subjects arrived at 9:30 AM. Following instrumentation, subjects remained supine for 30 min to acclimate. After acclimation, at least 10 min of continuous baseline data were recorded. With completion of supine measurements, 70° HUT testing began. Tilt-testing continued for a maximum of 10 min. All subjects in both the POTS and control groups finished the full 10 min tilt test without any adverse events.

### Data analysis

Data were measured continuously and synchronously. Signals were converted with an analog-to-digital converter (DI-720 DataQ Ind, Milwaukee, WI) connected to a desktop computer and analyzed offline. Following tilting, the first minute of data were omitted from analysis until each subject stabilized; this time contained a transient period of “initial orthostatic hypotension” (Wieling et al., 2007) from which all subjects recovered. We obtained averaged data (HR, BP, CO, TPR, ETCO2, CBFv) for each RR-interval and initially averaged data by time epochs: baseline, 1–2, 2–4, 4–6, 7–10 min. However, upright data averages did not vary with time for any given measurement and therefore upright data were expressed as averages over all upright epochs. Baseline and tilted MAP autospectra, mean CBFv autospectra, and transfer function analyses were reported as averages over the following frequency bands: very low frequency (VLF) = 0.01–0.04Hz, low frequency (LF) = 0.04–0.13 Hz, high frequency (HF) = 0.13–0.4Hz. In addition we reported MAP and CBFv peak frequency and bandwidth for baseline and tilted conditions. The term power within a band is used to describe the autospectral power summed over frequencies falling within a given frequency band.

For calculation of the transfer function, data was preprocessed by discrete wavelet smoothing using Dabauchies least asymmetric (LA12) mother (generating) wavelet function (Daubechies, 1988). The mother wavelet generated a dyadic orthonormal basis and multiresolution analysis was used to retain signals from 0.0078 Hz through 0.4 Hz. We employed an extended version of the discrete wavelet transform described by Percival and Walden to produce a maximal overlap discrete wavelet transformation which fills all time points at each scale, allows precise alignment of the signal and its wavelets, allows for any sample size, and has zero phase-shifted details (Percival and Walden, 2000). The signal was detrended by removing the smoothed signal residua obtained from the scaling function.

Thereafter, OAP and OCBF autospectra, and cross-spectral density were obtained from preprocessed signals using Welch's method (Welch, 1967) with overlapping intervals lasting 128 s. The lowest detectible frequency was then 1/128 = 0.0078 Hz. Additional transfer function analysis followed the methods of Zhang et al. (1998). The strength of a linear relation between OAP and OCBF was quantitated by the squared coherence function, defined as the ratio of squared cross spectrum divided by the product of the OAP and OCBF power spectra (Zhang et al., 1998). The transfer function was derived as the ratio of the cross spectral density to the MAP autospectrum (Lyons, 2011). Gain was defined as the magnitude or amplitude of the transfer function at a given frequency while phase difference was defined by the phase of the transfer function. The transfer function was computed as averages over frequency bands: VLF, LF, HF. Autospectral powers were also expressed in bands. We also examined the fine structure of the autospectra to obtain peak frequency and bandwidth OAP and OCBF.

## Results

### Supine

#### Hemodynamic data

Hemodynamic data for control subject and POTS patients are shown in Table 1. There was no significant difference in SBP, DBP or MAP, ETCO2, cardiac output, TPR or mean CBFv in POTS compared to control subjects. There was a significantly higher HR in POTS compared to control subjects (*P* < 0.01).

**Table 1:** **Hemodynamic parameters measured supine and upright in control subjects and in patients with Postural Tachycardia Syndrome (POTS)**.

	**Supine**		**70° HUT**	
**Measurement**	**POTS**	**Control**	**POTS**	**Control**
SBP (mmHg)	121 ± 7	123 ± 4	135 ± 7	128 ± 6
DBP (mmHg)	60 ± 3	61 ± 2	74 ± 3^*^^‡^	65 ± 3
MAP (mmHg)	78 ± 5	81 ± 2	95 ± 5^*^^‡^	86 ± 2^*^
HR (bpm)	80 ± 6^‡^	61 ± 2	125 ± 8^*^^‡^	85 ± 4^*^
ETCO_2_ (mmHg)	43 ± 1	43 ± 1	39 ± 1^*^	40 ± 1^*^
CO (L/min)	5.6 ± 0.4	5.5 ± 0.5	4.6 ± 0.6^*^	5.2 ± 0.2
TPR(mmHg/L/min)	15.5 ± 1.8	14.9 ± 0.9	20.0 ± 2.0^*^	18.4 ± 1.4^*^
Mean CBFV (cm/s)	69 ± 6	75 ± 3	64 ± 5^*^	70 ± 4^*^

SBP, systolic blood pressure; DBP, diastolic blood pressure; MAP, mean arterial blood pressure; HR, heart rate; ETCO_2_, end tidal CO_2_; CO, cardiac output; TPR, total peripheral resistance; Mean CBFV, mean cerebral blood flow velocity.

‡ p < 0.05 comparing POTS to control;

* p < 0.05 comparing upright to supine.

#### Autospectral power

Autospectral data (MAP and CBFv variability data) and transfer function analyses are presented in Table 2. There were no differences in mean arterial pressure variability either total or divided among VLF, LF, and HF bands. While VLF and HF cerebral blood flow velocity power did not differ for POTS compared to control subjects, LF CBFv power was significantly larger (*P* < 0.05) for POTS.

**Table 2:** **Mean arterial pressure → Cerebral blood flow velocity transfer function analysis and autospectral peaks and bandwidths in control subjects and POTS patients measured Supine and during 70° head-up tilt**.

	**Supine**		**70° HUT**	
	**POTS**	**Control**	**POTS**	**Control**
MAPVar	8.4 ± 2.1	6.7 ± 1.4	24.3 ± 4.1*^‡^	11.8 ± 3.3
MAPV—VLF	4.6 ± 1.2	3.4 ± 0.7	4.5 ± 0.7	5.4 ± 1.1*
MAPV—LF	3.9 ± 0.4	2.6 ± 0.6	18.4 ± 4.1*^‡^	8.8 ± 2*
MAPV—HF	0.4 ± 0.1	0.3 ± 0.1	2.2 ± 0.6*	2.0 ± 0.6*
CBFvVar	10.1 ± 1.1	10.3 ± 1.3	29.3 ± 4.7*^‡^	14.7 ± 2.6
CBFvVar—VLF	4.0 ± 0.7	5.6 ± 1.1	3.7 ± 0.9	5.4 ± 1.2
CBFvVar—LF	4.3 ± 0.4^‡^	2.6 ± 0.6	22.1 ± 2.7*^‡^	6.7 ± 1.2*
CBFvVar—HF	0.8 ± 0.2	1.3 ± 0.4	2.2 ± 0.6*	2.2 ± 0.6*
VLF coherence	0.18 ± 0.07	0.23 ± 0.09	0.12 ± 0.02	0.13 ± 0.05
VLF gain (mmHg/cm/s)	0.43 ± 0.07	0.57 ± 0.12	0.27 ± 0.04*^‡^	0.44 ± 0.08
VLF phase (degrees)	−42 ± 18	−64 ± 19	−28 ± 11	−34 ± 16
LF coherence	0.81 ± 0.04^‡^	0.61 ± 0.08	0.96 ± 0.01*^‡^	0.80 ± 0.04*
LF gain (mmHg/cm/s)	0.78 ± 0.04	0.88 ± 0.12	1.51 ± 0.09*^‡^	0.86 ± 0.12
LF phase (degrees)	−41 ± 15	−31 ± 16	−14 ± 4*^#^	−25 ± 10
HF coherence	0.65 ± 0.11	0.54 ± 0.11	0.77 ± 0.08	0.63 ± 0.11
HF gain (mmHg/cm/s)	0.96 ± 0.13	1.18 ± 0.14	0.86 ± 0.08	0.77 ± 0.13*
HF phase (degrees)	−41 ± 14	−28 ± 15	−23 ± 7	−18 ± 12
MAP peak frequency (Hz)	0.052 ± 0.011^‡^	0.034 ± 0.008	0.091 ± 0.004*^‡^	0.071 ± 0.008*
MAP bandwidth (Hz)	0.044 ± 0.004	0.033 ± 0.004	0.032 ± 0.005*^‡^	0.056 ± 0.010*
CBFv peak frequency (Hz)	0.064 ± 0.012	0.071 ± 0.021	0.092 ± 0.002*^‡^	0.077 ± 0.006
CBFv bandwidth (Hz)	0.041 ± 0.011^‡^	0.063 ± 0.019	0.034 ± 0.004^‡^	0.060 ± 0.010

*p* < 0.05 comparing POTS to control;

‡ p < 0.05 comparing upright to supine.

MAPVar, Mean Arterial Pressure Variability in the Very Low (VLF), Low (LF) and High (HF) frequency bands.

CBFvVAR, Cerebral Blood Flow Variability in the Very Low (VLF), Low (LF) and High (HF) frequency bands.

#### Autospectral peaks and bandwidths

The frequency at peak MAP power was significantly larger for POTS (*P* < 0.05), but there was no difference in the frequency at peak CBFv power. Bandwidths for MAP were not different, but were decreased for CBFv (*P* < 0.05).

#### Transfer function analysis

Coherence, gain and phase were not different at VLF or HF for POTS compared to control. Also, gain and phase were not different for LF while coherence was increased (*P* < 0.01) for POTS. Note that VLF coherence was always less than 0.5, implying either no relationship, a missing interacting term, a non-linear relationship, or the presence of excessive noise (Taylor et al., 1998). Typically gain and phase data are regarded as unreliable linear estimates under these circumstances.

### Upright

#### Hemodynamic data

Table 1 shows that during HUT, SBP was not different for POTS or control but DBP was increased in POTS compared to supine (*P* < 0.01) and in control compared to supine (*P* < 0.025). MAP was increased compared to supine in POTS (*P* < 0.01) and in control (*P* < 0.025). Upright MAP was significantly larger in POTS compared to control (*P* < 0.05). HR was increased in both POTS and control subjects (*P* < 0.001) but was also significantly larger in POTS than control (*P* < 0.001). ETCO_2_ decreased modestly with upright tilt for both groups (*P* < 0.05). Cardiac output was significantly reduced in POTS compared to supine (*P* < 0.05), and TPR increased for both groups compared to supine (*P* < 0.001). CBFv during HUT was reduced (*P* < 0.025) compared to supine in both POTS and control but did not differ upright between groups.

Figure 1 shows representative tracings of phasic BP and CBFv in the time domain, in control subjects and POTS patients, while supine during HUT. With upright tilt, oscillations in arterial pressure intensify in POTS. Upright oscillations are much less striking in control and occur at a lower frequency. Oscillations in arterial pressure are translated into large synchronous oscillations in CBFv in POTS patients.

#### Autospectral power

***MAP data..***There were large differences in total mean arterial pressure variability (MAPVar) and MAPVar dispersed among VLF, LF, and HF bands, as shown in Table 2. Overall MAP variability was markedly increased in POTS compared to supine or compared to upright control data (*P* < 0.001). VLF power did not change with tilt in POTS but increased slightly in control subjects (*P* < 0.05). LF power increased for control (*P* < 0.01) and for POTS (*P* < 0.001) compared to supine but was markedly larger (*P* < 0.001) in POTS compared to control when upright. HF variability was similarly increased for POTS and control (*P* < 0.01).

***CBFv data..***There were large differences in total CBFv variability and CBFv dispersed among VLF, LF, and HF bands. Overall CBFv variability was markedly increased in POTS compared to supine or to upright control data (*P* < 0.001). While VLF cerebral blood flow velocity power did not differ for POTS compared to control subjects, LF power was significantly larger (*P* < 0.001) for POTS compared to controls when upright. LF power increased above supine for both POTS and controls, however. Finally, HF power increased similarly with tilt for both POTS and control subjects (*P* < 0.05).

#### Autospectral peaks and bandwidths

**Figure 2:**
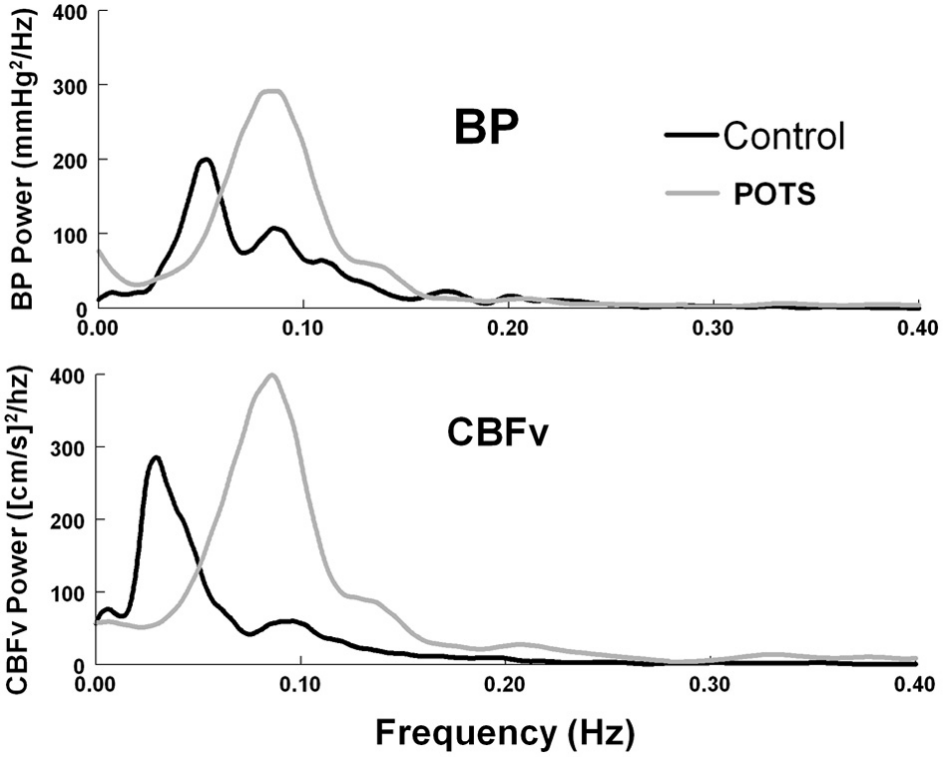
**Autospectral curves showing mean arterial pressure (BP) power and cerebral blood flow velocity (CBF_*v*_) power, comparing control subjects to POTS patients while upright**. There is a significant shift to higher frequencies in POTS patients (*p* < 0.01).

Peak MAP power and CBFv power were shifted to higher frequencies for POTS and controls (*P* < 0.01). Peak frequency in POTS was significantly higher than peak frequency in control subjects (*P* < 0.001) and the peaks of MAP and CBFv were similar within each group, but there was no difference in the frequency at peak CBFv power. Bandwidths for MAP and CBFv were smaller in POTS upright compared to supine and smaller than upright control. This is depicted in the autospectra curves in Figure 2 for representative upright POTS and control subjects; differences in upright frequency peaks and in the dispersion of spectral power are apparent.

#### Transfer function analysis

Very low frequency gain was lower in POTS compared to supine or to upright control (*P* < 0.01). However, because coherence remained very low, there can be no real importance attached to this finding. LF coherence was increased for POTS (*P* < 0.001) and control (*P* < 0.01) and was significantly larger in POTS (*P* < 0.01). LF gain was increased and phase was decreased in POTS (*P* < 0.001) compared to supine. LF gain and phase were unchanged from supine in control subjects. HF coherence and phase did not differ from supine in both groups but gain was significantly smaller (*P* < 0.05) in control subjects.

The main findings are that both MAP and CBFv spectra shift to a high frequency in control and POTS when upright, but with a larger shift in POTS. MAP and CBFv autospectral maxima in POTS occur at the same upright frequency and are highly coherent with reduced phase difference and increased gain. These data indicate reduced dynamic cerebral autoregulation in POTS (Diehl et al., 1995; Panerai, 1998; Tzeng et al., 2012).

**Figure 3:**
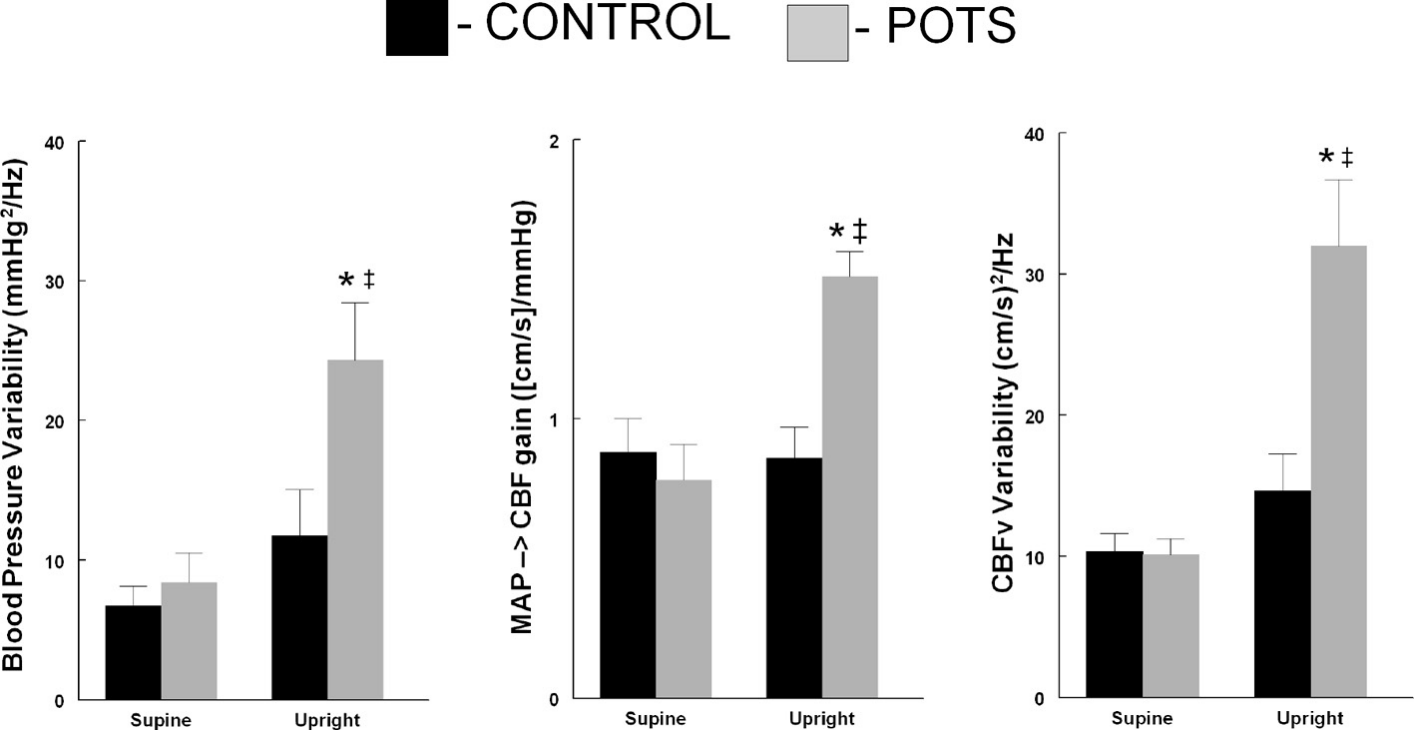
**Comparison of blood pressure variability (left panel), low frequency gain (Middle Panel) and cerebral blood flow velocity variability (right panel comparing control subjects (■) to POTS patients (□) supine and upright during 70° head-up tilt**. ^*^ *p* < 0.05 comparing POTS to control; ^‡^ *p* < 0.05 comparing upright to supine.

Taken together the data demonstrate the marked increase in oscillatory cerebral blood flow power in POTS. Increased OCBF is in part due to increased OAP and in part due to increased transfer function gain over frequencies of maximum oscillatory blood pressure power. These differences are depicted in Figure 3.

## Discussion

Oscillations of cerebral blood flow (more precisely oscillatory cerebral blood flow velocity) are greatly increased in POTS compared to control subjects when upright. While this observation has been reported previously in POTS, one study described subjects with and without orthostasis-induced hyperpnea (Schondorf et al., 2005), while the other study evaluated neurocardiogenic syncope patients with excessive upright tachycardia attributed to POTS (Diehl et al., 1999). Our study is unique as it only enrolled POTS patients who did not exhibit orthostatic hyperpnea/hypocapnia. Our findings are the result of the combined effects of increased oscillatory arterial pressure in the low frequency band and increased LF transfer gain from oscillatory arterial pressure to oscillatory cerebral blood flow velocity.

### Increased OCBF implies reduced cerebral autoregulation in POTS

Cerebral autoregulation comprises properties of the cerebral vasculature that, in the absence of large environmental or metabolic changes, maintain overall cerebral blood flow relatively unchanged despite changes in blood pressure (Panerai, 1998). Brain blood flow is closely regulated because of the critical dependence on oxygen and substrate delivery. Increased BP that might otherwise cause potentially injurious increases in CBF is counter-regulated by cerebral vasoconstriction and vice versa; this represents the autoregulation paradigm. Cerebral autoregulation is usually described as being static and dynamic: static refers to changes in mean CBF; dynamic refers to ongoing time dependent changes in CBF during AP. However, a true constant CBF unchanged by AP exists more in concept than in reality. There are several approaches to the evaluation of cerebral autoregulation (Zhang et al., 1998; van Beek et al., 2008; Tzeng et al., 2012), among them Fourier methods most suitable for linear time-invariant systems. An input-output relationship is presumed between AP, acting as the input or motive force and cerebral blood flow the output which takes the form of a convolution integral in the time domain. Often, the relationship between AP and CBF are more easily calculated in the frequency domain in which the convolution operation is replaced by multiplication of the Fourier transform of CBF by the transfer function, which is a function of frequency, to obtain the Fourier transform of AP.

The properties of the transfer function are encapsulated by parameters of gain, phase and coherence at each frequency. Using these, investigators have demonstrated that cerebral autoregulation is most effective at lower frequencies ≤0.1 Hz while at higher frequencies the system functions as a high pass filter (Claassen et al., 2009). Thus, for example, changes in AP at the frequency corresponding to heart rate are transmitted to CBF. However, these large amplitude, high frequency oscillations in CBF are essentially damped out at the tissue level so only lower frequency oscillations penetrate to the microvasculature, as shown by NIRS studies (Li et al., 2013). OCBF is less dependent on OAP at lower frequencies, which defines the operational frequency range for autoregulation. Applied to transfer function analysis, low gain (amplitude of OCBF divided by amplitude of OAP), lower coherence and increased phase difference between OAP and OCBF indicate effective autoregulation because AP and CBF are then relatively independent. On the other hand, high gain, higher coherence, and decreased phase difference represent ineffective autoregulation because AP and CBF change in synchrony, as we show here for POTS patients while upright. Our finding of ineffective cerebral autoregulation in POTS is in contrast to previous studies (Diehl et al., 1999; Schondorf et al., 2005), which concluded that cerebral autoregulation was not different in POTS compared to controls. These discrepancies may be due to differences in the characteristics of the study groups; one was likely composed of those with and without hyperpnea/hypocapnia (Schondorf et al., 2005) while the other enrolled syncopal patients with POTS (Diehl et al., 1999).

#### OCBF is driven by OAP and is not spontaneous vasomotion

The term “spontaneous cerebral blood flow oscillations,” has been used to describe supine OCBF with maximum power at low frequencies (Obrig et al., 2000; Schytz et al., 2010). “Spontaneous” presupposes independence of AP or HR, and therefore caused by spontaneous vasomotion (Haddock and Hill, 2005). Vasomotion comprises fluctuations in smooth muscle contraction and vascular diameter that depend only on extracellular calcium (Giller et al., 1999). Indeed, very low frequency oscillations between 0.01 and 0.03Hz are also present in AP and CBF but are not correlated with one another. These oscillations could represent spontaneous vasomotion at similar frequencies because vasomotion should be common to systemic arterial and cerebral vasculature. Nonlinear VLF coupling is also possible (Zhang et al., 2002). Similar oscillations are described in studies employing BOLD fMRI (Biswal et al., 1997). During orthostasis, very low frequency OCBF almost entirely give way to low frequency OCBF in the range of 0.07–0.13 Hz (Ocon et al., 2011) in POTS patients, which are coupled to oscillations in AP (Zhang et al., 2000).

OAP has been appreciated for more than a century, and are often referred to as “Mayer waves” (Julien, 2006). Mayer waves may take origin in peripheral vasomotion, but roles for CNS oscillators and by time-delay in intact sympathetic arterial baroreflex feedback loops have also been proposed (Guyton and Harris, 1951; Kawada et al., 2000; Hammer and Saul, 2005). It is therefore interesting that sympathetic baroreflex activity is enhanced during orthostasis, particularly in POTS patients (Bonyhay and Freeman, 2004; Muenter et al., 2005) in part as a result of central hypovolemia resulting from decreased venous return (=CO), as we and others have demonstrated. Such enhanced sympathetic activity should increase OAP; this conjecture finds support by the finding of close coupling of OCBF to OAP over a relatively narrow bandwidth corresponding to the Mayer wave frequency.

Increased OAP and increased gain markedly increases OCBF. The bandwidth is broader in control subjects and centered at lower frequencies. Close coupling of OCBF to OAP indicates impaired cerebral autoregulation, i.e., OCBF is almost completely dependent on OAP (Lassen, 1959). This form of “dynamic” oscillatory cerebral autoregulatory impairment is found in all of our POTS patients (Ocon et al., 2009).

Like cerebral neurocognitive impairment, these OCBF findings are reliably present when upright in POTS patients but absent in control subjects. While it is not presently possible to assign a causal connection, it might be justified to regard such findings as consistent with OCBFv as a marker or correlate of this impairment, often described as brain-fog (Ocon et al., 2009). While it is difficult to show statistical association between phenomena (OCBF and brain-fog) which are always present, we will need to demonstrate that decreasing OCBFv improves executive working memory or the converse using objective measures such as N-back testing.

We have found consistent increased upright OCBF in POTS that might serve as a marker of cognitive deficits in POTS. The effects of OCBF on neuronal functioning remains to be determined, however, this close coupling of CBF to MAP indicates impaired cerebral autoregulation that may underlie upright neurocognitive dysfunction in “normocapneic” POTS patients as investigated here, and perhaps in hypocapneic, hyperpneic POTS patients as well.

## Limitations

Transfer function analysis uses linearized spontaneous fluctuations in AP and CBFv to obtain information. The linear hypothesis is at best an approximation but still provides useful information. An additional drawback is the relatively small range of amplitudes of OAP and OCBFv. However, since POTS is not associated with large changes in arterial pressure, if there were a causal relationship between AP, CBF, and cognitive impairment, it would depend on relatively small ranges of oscillatory amplitudes.

TCD measures oscillatory cerebral blood flow velocity (OCBFv) rather than oscillatory cerebral blood flow (OCBF). OCBF is dependent on the diameter of the insonated artery. MCA diameters may be resistant to change during orthostatic stress (Serrador et al., 2000). Oscillatory frequencies of OCBFv and corresponding OCBF are likely the same and equal to the frequency of OAP and phase relationships should also be similar. In general (Liu et al., 2013) even under conditions of changing BP, cerebral autoregulation can be assessed by TCD, but may be underestimated.

TCD only measures blood flow through a particular cerebral blood vessel but has good temporal accuracy. The MCA was used because it is the main vessel that perfuses the area of the brain activated during previous executive memory testing (Ross et al., 2013). TCD does not have regional accuracy, and therefore the values of CBFV obtained may reflect an average over areas perfused. Perfusion could vary with brain location during orthostasis. However, such variations are small l (Deegan et al., 2010) and it is unlikely that directionally opposite changes in blood flow would occur as a function of geography. We did not measure TCD in both hemispheres as previous work showed that MCA CBFv was not different between the hemispheres during orthostatic stress (Ocon et al., 2012).

## Author contributions

Marvin S. Medow made substantial contributions to the design, acquisition, analysis and interpretation of the work and drafting the work; Andrew T. Del Pozzi, Zachary R. Messer, and Courtney Terilli made substantial contributions to the acquisition of the work, drafting and revising it critically for intellectual content and final approval of the version to be published and Julian M. Stewart made substantial contributions to the conception and design of the work, analysis, and interpretation of data, drafting the work and final approval of the version to be published.

### Conflict of interest statement

The authors declare that the research was conducted in the absence of any commercial or financial relationships that could be construed as a potential conflict of interest.
